# Recent Advances and Applications of Experimental Technologies in Marine Natural Product Research

**DOI:** 10.3390/md13052694

**Published:** 2015-04-29

**Authors:** Ke Li, Yu-Wen Chung-Davidson, Ugo Bussy, Weiming Li

**Affiliations:** Department of Fisheries and Wildlife, Michigan State University, Room 13 Natural Resources Building, 480 Wilson Road, East Lansing, MI 48824, USA; E-Mails: like4@msu.edu (K.L.); chungyuw@msu.edu (Y.-W.C.-D.); bussy@msu.edu (U.B.)

**Keywords:** marine organism, sampling strategies, nanoscale NMR, computational chemistry

## Abstract

Marine natural products are a rich source of novel and biologically active compounds. The number of identified marine natural compounds has grown 20% over the last five years from 2009 to 2013. Several challenges, including sample collection and structure elucidation, have limited the development of this research field. Nonetheless, new approaches, such as sampling strategies for organisms from extreme ocean environments, nanoscale NMR and computational chemistry for structural determination, are now available to overcome the barriers. In this review, we highlight the experimental technology innovations in the field of marine natural products, which in our view will lead to the development of many new drugs in the future.

## 1. Introduction

The ultimate goal of natural product research is to discover promising candidate drugs for treatment of human diseases [1,2]. Natural products, because of their coevolution with biological targets, are good starting points for drug discovery [3,4,5]. Overall, natural products are characterized as structurally complex molecules possessing a well-defined spatial orientation. These compounds are potentially new active pharmaceutical ingredients. Indeed, approximately half of the new drugs released so far have been discovered or designed based on the chemical structures elucidated from natural products [1], and half of the 20 best-selling medicines are related to natural products [6]. Currently, natural product-derived therapeutics are mostly developed from compounds of terrestrial origins [7].

**Figure 1:**
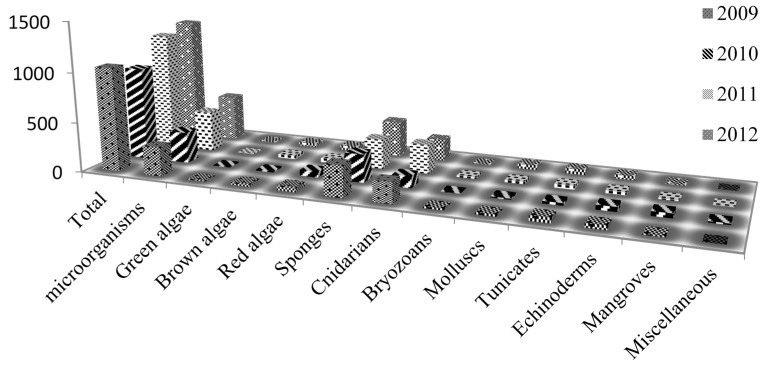
Detailed biogenetic origins of novel marine natural products from 2009 to 2012, *y*-axis represents the number of compounds.

Recently, the research focus for natural products has been shifted from terrestrial to marine sources because of the superior chemical and biological novelties of marine compounds [8,9]. In general, natural molecules isolated from marine environment show higher and more significant bioactivity than those from terrestrial environment. In cytotoxicity screenings, approximately 1% of the tested marine samples showed anti-tumor potentials *versus* 0.1% of the tested terrestrial samples [10]. In addition, 71% of the molecular scaffolds reported in the Dictionary of Natural Products were exclusively utilized by marine organisms [11]. Blunt *et al*. (Figure 1) reviewed and summarized the progress in marine natural products characterized from 2009 to 2013 [12,13,14,15,16], and listed almost all the novel marine natural compounds discovered during that time period. However, the emerging new techniques that promote the isolation and identification of these marine natural products were not discussed in detail. In this review, we attempt to fill this gap by summarizing these emerging techniques.

Marine natural product research faced several challenges, ranging from limited access to the marine environment to chemical and biological characterizations of promising natural product. In particular, the difficulties in sample collection and consequently the limited amounts of active materials in marine organisms render the isolation or identification (or both) of the target molecules fastidious. Nevertheless, advancement in sampling techniques and structural determination strategies have played crucial roles in recent progress of marine drug discovery. Furthermore, computation-assisted structural elucidation for molecules with complex structures have also found great utility.

## 2. Extreme Environment Sampling Techniques

Sampling in the ocean requires specific technique and sophisticated equipment. This hurdle has been the main reason for the limited exploration of natural products in the oceanic environment. In general, the access capacity of manned submersibles and trawling operations to different levels will be the greatest influence on any regional analysis, rather than a limitation only occurred in the geographical distribution of marine fauna (and their natural products) in deeper water. Interestingly, the origin of deep submersible was motivated by biologist William Beebe, who observed fish and other diverse forms of deep sea life with the naked eye in 1934 [17]. After several decades, human occupied deep submergence vehicle, *SHINKAI* 6500, was launched in 1991, and was capable of diving to the maximum depth of 6500 m. This submersible has produced a number of impressive achievements in research on the topography and geology of the ocean floor, as well as life inhabiting the deep seas [18]. Because of the cost and risk, human occupied vehicles cannot be used in research at depths greater than 6500 m [17]. Unoccupied vehicles are the optimum alternative for investigation of the ocean floor. So far, *KAIKO* is one of the deepest diving devices which is capable of diving to a maximum depth of 10,000 m and surveying deep ocean areas where it is impossible and too dangerous for human occupied submersibles due to the complicated topography [19]. The newest model *KAIKO* 7000 was created by a 7000 m class optical fiber cable-type remotely operated vehicle integrated with *KAIKO*’s launcher. In search of marine natural products, research-class deep-sea sampling equipment, expanded access to submersibles, remotely operated vehicles, and trawling technology through collaboration with deep-sea industries are enabling researchers to further explore deep-sea fauna and their natural products [20,21].

Since 2009, more than 4400 novel marine natural compounds have been identified [12,13,14,15,16]. However, only 4% (188) of them were isolated from deep-water (50 to 5000 m) marine fauna, including *Bryozoa*, *Chordata*, *Cnidaria*, *Echinodermata*, *Mollusca*, *Porifera*, and microorganisms, owing to the drastic environmental conditions. Amazingly, 75% of these compounds of deep-water origin displayed bioactivity, and almost half of them exhibited low micromolar cytotoxicity toward a range of human cancer cell lines. As an example, a deep-sea natural product-derived drug (eribulin mesylate) has already been released in the market. Moreover, the number of potentially anti-cancer microbes significantly increased along the depth of the investigated oceanic environment [22]. The higher incidence of bioactive natural products isolated from deep-sea organisms has been an impetus for scientists and engineers to pursue this line of research by developing and improving high performance sampling techniques.

**Figure 2:**
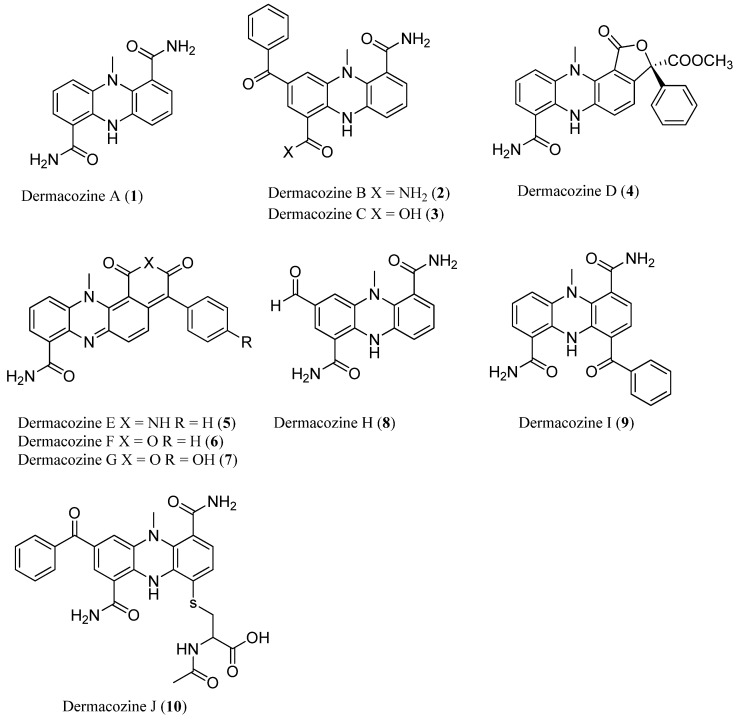
Dermacozines A–J (**1**–**10**).

To date, natural products isolated from the deepest ocean sediment (10,898 m deep) include ten novel and cytotoxic compounds dermacozines A–J (**1**–**10**) (Figure 2) [23,24], isolated from marine bacterium *Dermacoccus abyssi,* initially obtained from the Philippine Sea. Since 1997, ocean sediments have been collected by the distance-controlled submarine *KAIKO*, using sterilized mud samplers [25]. As a result, Pathom-aree *et al*., succeeded in discovering 38 novel actinomycete strains [26], providing a precious resource to marine natural product researchers.

**Figure 3:**
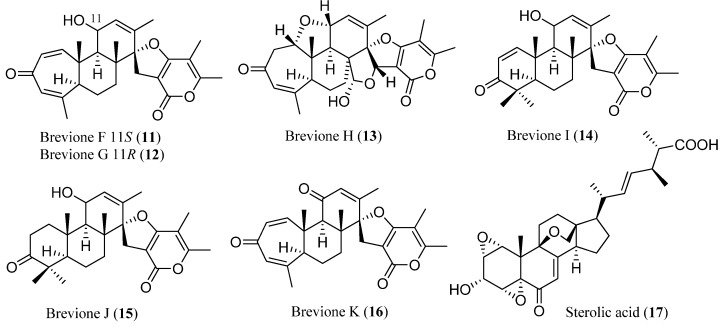
Breviones F–J (**11**–**16**) and sterolic acid (**17**).

Deep-sea bacteria, producing over 25% of the natural products from deep-sea organisms reported so far, have been cultured from sediment obtained from 10,898 m below water. In addition, deep-sea fungi, from which over 17% of the reported metabolites have been isolated, have been cultured from sediment collected from 5115 m depth [22]. From the crude extract of *Penicillium* sp. obtained from this sediment, Che and co-workers isolated six novel bioactive spiroditerpenoids breviones F–K (**11**–**16**) (Figure 3) and a polyoxygenated sterol, sterolic acid (**17**) (Figure 3), among which brevione I (**14**) showed significant cytotoxicity [27,28]. The deep-water sediment sample (5115 m) was collected in the East Pacific Ocean (145°2′W, 07°37′N), in September 2003. Unfortunately, further details for collection method of this sediment are unavailable.

**Figure 4:**
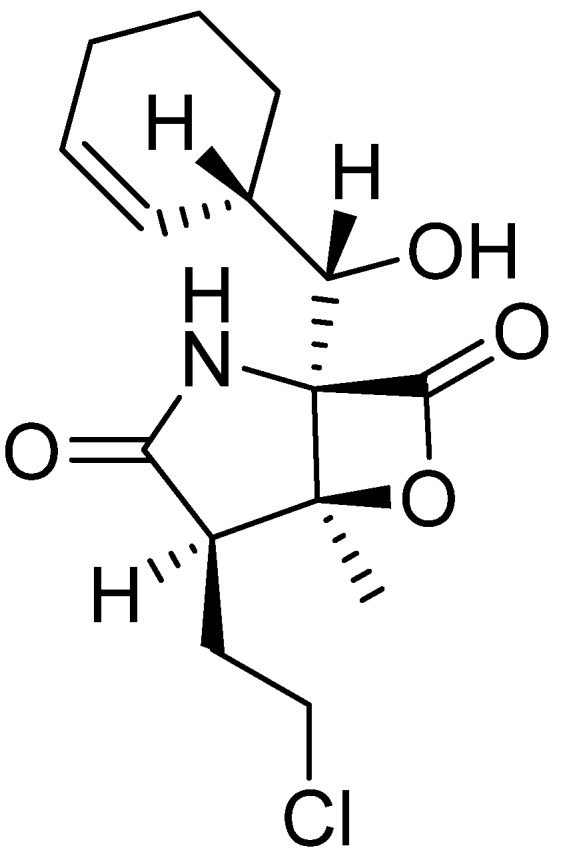
Salinosporamide A (**18**).

Fenical and co-workers’ studies reveal that the metabolite profile of actinomycetes derived from near shore sediment samples show high similarity with that of strains previously observed from land [29]. They succeeded in discovering potent proteasome inhibitor salinosporamide A (**18**) (Figure 4) from phylogenetically novel marine actinomycete strains *Salinispora tropica*, which was isolated from sediment samples collected at a depth of about 1 m from the mangrove environment [30]*.* To further investigate biological diversity, it has become a priority to access oceanic, deep-sea sediments to determine whether there is a higher probability that new marine actinomycete taxa will be recovered from these sites than from near-shore sites. They modified a small-scale tethered sediment grab coupled with the use of an electric-powered fishing reel, providing reliable access to marine sediments at depths of 1500 m. However, these techniques have a defect because they leave large portions of the ocean bottom out of sampling range. Finally, they were able to design a device to obtain samples from the bottom of the ocean, with the depth exceeding 2000 m, using relatively small boats. These techniques possess an autonomous bounce corer that can be deployed by hand.

In addition to the deep ocean, Polar Regions are considered huge reservoirs of diverse microorganisms. However, few studies have been performed on the antimicrobial activities of isolates from Antarctic soils [31], seawater and sponges [32,33,34]. The aim for Antarctic bacteria study was to identify special microorganisms with the ability to produce cold-active antimicrobial compounds with potential for chilled food preservation. From Antarctic soils 13 bacteria of 4492 colonies (detection rate 0.29%) were confirmed as growth-inhibitor producers [31]. From Antarctic seawater, 21 of 140 bacteria (15%), predominantly Actinobacteria, exhibited antagonistic properties against marine bacteria of Antarctic origin [32]. The aerobic heterotrophic bacterial communities isolated from three different Antarctic sponge species were analyzed for their ability to produce antimicrobial compounds active toward Cystic Fibrosis opportunistic pathogens belonging to the *Burkholderia cepacia* complex (Bcc) [34]. The data in this study revealed that most of these sponge-associated Antarctic bacteria, belonging to different genera, were able to completely inhibit the growth of bacteria belonging to the Bcc.

In summary, advanced diving, voyage, and survey technologies provide unprecedented access to a previously untapped source of biodiversity, and hence a potential source for marine natural products or drug candidates. However, one concern is that the artificial fermentation environment used for these deep-ocean microbes is very different from their native marine conditions, which may lead to unexpected biochemical reactions [35]. In addition, symbiotic interactions between bacteria and animals may result in defensive chemicals for the host and nitrogen supply for the bacteria. Therefore, without interaction with the host, the bacteria fermented in the medium broth may produce chemicals different from their natural metabolites.

## 3. Hyphenated Techniques for Structural Determination at the Nanomole Scale

Modern research for marine natural products has relied heavily on fractionation based on the combination of a valid fractionation by a high-resolution separation technique and detection systems possessing high information density. Combining different instrumentations as hyphenated devices, such as CE-MS (capillary electrophoresis-mass spectrometry), HPLC-MS (high performance liquid chromatography-mass spectrometry) or HPLC-NMR (high performance liquid chromatography-nuclear magnetic resonance) for separation and detection, is one of the most active research fields in modern analytical chemistry. These techniques play a predominant role in natural product discovery related fields such as phytochemistry or pharmacognosy. Seger *et al.* reviewed modern high-end detectors (Mass spectrometry and NMR spectroscopy) for high-resolution separation techniques (HPLC and capillary electrophoresis (CE)), with a focus on publications from 2005 to 2013 [36]. This review highlighted the dramatic technological development of the past decade, including the introduction of stable and reliable QqQ tandem mass spectrometers that have become workhorses in quantitative HPLC-MS/MS analyses, the invention of novel high-resolution Fourier transform mass spectrometers (*i.e*., the “orbitrap”) and steady improvements in high-resolution Time of Flight (ToF) mass spectrometers, both of which are utilized in qualitative HPLC-MS/MS assays, as well as the maturation of HPLC-NMR by the development of the HPLC-SPE-NMR platform. At the present time, structure elucidation of secondary natural products in solution can only be achieved by the exhaustive use of 1D and 2D homo- and heteronuclear NMR measurements assisted by other spectroscopic methods, including high resolution MS. Mass spectrometry, even the tandem mass spectrometers with high resolution mass selector technologies, can hardly provide more information than the molecular formula. The limited possibility to elucidate the structure of unknown analytes, not to mention their 3D structures, from the HPLC-MS data has been recognized as major methodological bottleneck.

**Figure 5:**
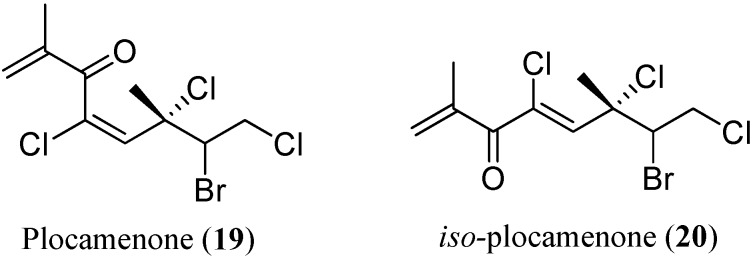
Plocamenone (**19**) and *iso*-plocamenone (**20**).

Traditionally, HPLC and NMR have been the primary tools used to isolate and determine the structures of natural products of interest. Advances in probe design and suppression techniques facilitated the development of efficient protocols for HPLC-NMR, providing natural product chemists with a hyphenated technique that combines the two most powerful tools available in their field. HPLC-NMR has various modes of operation, with on-flow and stop-flow as the two primary modes. Sylvia and co-workers applied both on-flow and stop-flow HPLC-NMR to profile and identify metabolites from marine brown algae, red algae, and sponge [37,38,39,40,41]. The on-flow HPLC-NMR is a continuously evolving technique. It provides profiling information for the presence of structurally related secondary metabolites and is suitable for rapid identification of principal components. Recent advances in ultra-fast 2D NMR allow the acquisition of 2D spectra downstream of LC separation. Applying these techniques to natural product sciences facilitates the identification of the eluted compounds “on the flow”. The stop-flow HPLC-NMR analysis is then carried out to elucidate the structures of the eluents from the on-flow HPLC-NMR [36]. In the stop-flow mode, selected chromatographic peaks can be trapped in the HPLC-NMR flow cell for an indefinite period, thereby extending the acquisition time and consequently improving sensitivity and resolution in the resulting WET-1D ^1^H-NMR spectra [42]. Combining these two analyses, Sylvia *et al*. [40] succeeded in the identification of plocamenone (**19**), and a novel unstable double bond stereoisomer, *iso-*plocamenone (**20**) (Figure 5). Both plocamenone and *iso*-plocamenone showed elective antibacterial and moderate broad-spectrum antifungal activity [40].

The major advantage of on-flow HPLC-NMR is its ability to separate components *in situ* and real-time, hence it allows access to rich and mandatory structural information from NMR specific to every chromatographic signal. Compared to other analytical techniques, such as HPLC-MS and HPLC-Evaporative Light Scattering Detector (HPLC-ELSD), HPLC-NMR is a non-destructive technique that allows full recovery of all components for further analyses, such as bioassays or mass spectrometry. It is also a valuable analytical method for unstable compounds. The major drawbacks of HPLC-NMR are the inherently low sensitivity of NMR spectroscopy, the time constraints of the on-flow data acquisition, and the recurring NMR shift-referencing problem that occurs whenever solvent conditions changed. In addition, NMR is expensive, for both acquisition and maintenance of the instrument. Therefore, even though on-flow HPLC-NMR has improved upon the HPLC and NMR instrumentations, stopped flow and loop collection experiments are still widely applied in natural product chemistry and the pharmaceutical industry.

## 4. Nanomole NMR Techniques

**Figure 6:**
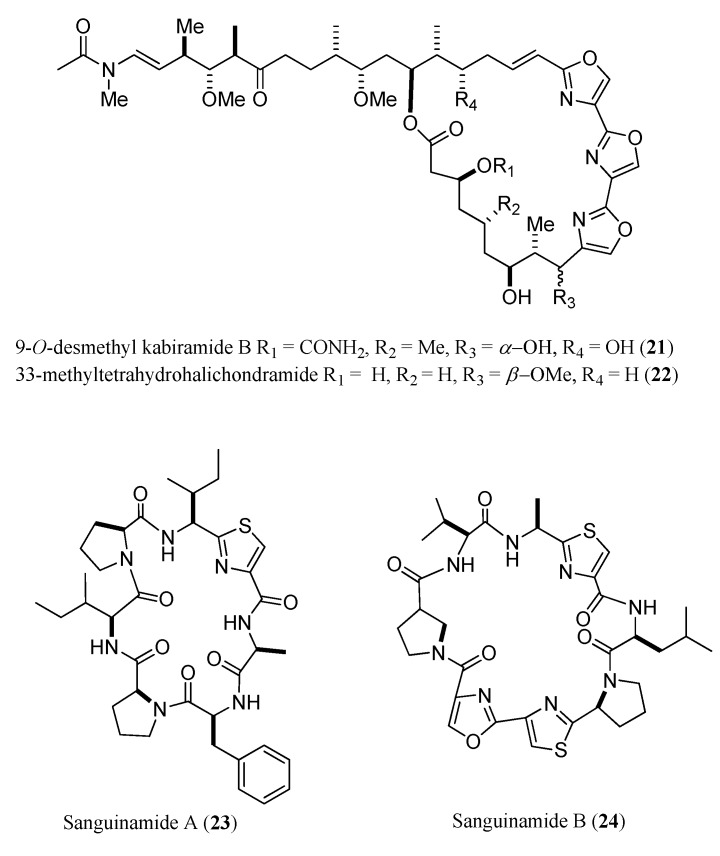
9-*O*-desmethyl kabiramide B (**21**), 33-methyltetrahydrohalichondramide (**22**), sanguinamides A (**23**) (0.39 mg) and B (**24**).

NMR spectroscopy has remained mandatory for absolute structural elucidation of organic analytes, despite tremendous improvement in the performances of mass spectrometry techniques. Marine natural product research was limited by the minute amount of purified compound, which seriously affected NMR performances. Recent advances in NMR structure elucidation methodologies have overcome this impediment. Meyer and coworkers reported characterization of picomole amounts of oligosaccharides by ^1^H-NMR spectroscopy with optimized sample preparation, water signal suppression, and instrument setup [43], further expanding the tremendous potential of NMR spectroscopy within the analytical arsenal available to natural product identification. In general, drastic improvements in NMR instruments, through low-volume tube probes and capillary probes, coupled with cryogenic cooled radiofrequency (rf) coils and preamplifier components, have led to an approximately 20-fold increase in sensitivity compared to conventional NMR instrumentation in the same field [44]. These instrumental improvements provide new perspectives for compound discovery from rare organisms, and for uncovering chemical complexity and diversity within single specimens [44]. Over the last decade, Molinski’s group applied nano-scale NMR techniques in marine natural products research, using 600 MHz NMR spectrometers with highly sensitive probes, a custom-built mm high-temperature superconducting (HTS) NMR cryoprobe (National High Magnetic Field Laboratory) and a commercial 1.7 mm cryoprobe [45]. This combination allowed full characterization of very minor components of *Hexabranchus sanguineus* [46] and *Phorbas* sp. [47,48,49]. As a result, two new trisoxazole analogues, 9-*O*-desmethyl kabiramide B (**21**) (0.62 mg, 0.04% w/w dry weight) and 33-methyltetrahydrohalichondramide (**22**) (1.3 mg, 0.07%), and two unexpected thiazole-containing peptides sanguinamides A (**23**) (0.39 mg, 0.023%) and B (**24**) (0.19 mg, 0.011%) (Figure 6) were fully characterized from submicromole samples by 1D and 2D NMR data in conjunction with MS and derivative correlation [46].

**Figure 7:**
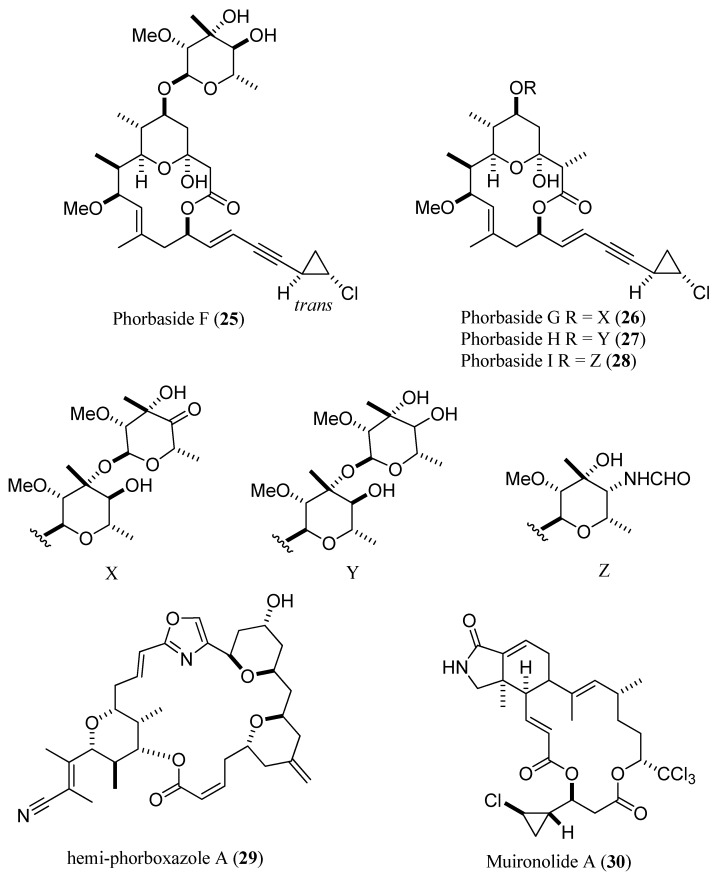
Phorbasides F–I (**25**–**28**), hemi-phorboxazole A (**29**), and muironolide A (**30**).

With a similar approach, six minute compounds, including phorbasides F–I (**25**–**28**) (F, 7.7 μg, [47]; G, 9.5 µg; H, 7.0 µg; I, 9.4 µg, [49]), hemi-phorboxazole A (**29**) (16.5 μg) [48], and muironolide A (**30**) (90 μg), [50] were fully characterized by MS, CD, and 2D NMR (COSY, ROESY, HSQC, HMBC) from one specimen of *Phorbas* sp. (Figure 7).

The leaps in nanomole NMR techniques have brought revolutionary improvements upon sensitivity in analyses of natural products. However, the high cost for maintaining low volume NMR micropryoprobes, NMR sample micro-tubes, and other consumables restricted the utility of nanomole NMR techniques. Apparently, nanomole NMR techniques are not mandatory for natural products obtained from large-scale plant extraction or microbial fermentations, which often provide tens to hundreds of milligrams of compounds. Nonetheless, the microcryoprobes NMR appears to be the only instrument capable of elucidating the structure of products at the nanomolar scale.

## 5. Computational Chemistry and Database

Structure determination is the most crucial step to achieve the goal of natural product research. The commonly used approaches, interpretation of MS and NMR data and stereochemical assignments by derivative reaction, have been greatly developed in the past several decades [51]. However, these techniques are time consuming. Alternatively, synthesis of the target molecule and subsequent comparison of their NMR spectroscopic data is another valuable and highly used approach. The restrictions for synthesis of natural products are (1) difficult to design reaction scheme; (2) very costly because the reaction material and/or experimental equipment requirement; and (3) time consuming due to the complicate synthesis scheme and quality control for each step. Nowadays, quantum mechanical calculations of NMR chemical shifts are an excellent tool for determining molecular structures [52,53,54,55] and apply to marine natural product research [56,57,58,59].

Quantum-chemistry methods have been continuously developed and improved by new theories and powerful computation. The continuous development of new computer facilities and computational methods allows the use of increasingly extended basic sets at either Hartree-Fock (HF), density functional theory (DFT), or post-HF methods, especially for atoms at the first two rows of the periodic table. These methods overcome the computational limitations, which are related to the size of the studied system and the accuracy of the theoretical approach. Notably, it has been reported that the use of DFT methods, with which relatively simple basic sets can yield accurate chemical shift predictions [60]. On the other hand, Goodman *et al*. [61] have reported that estimates of energy and isotropic shielding in solution by DFT methods can be reliably obtained by single-point calculations on the gas-phase of structures obtained from faster molecular mechanics conformational searches, thus circumventing the need for time consuming optimizations in solvent. In summary, DFT method plays a major role in predicting the chemical shift of molecules with multiple isomers, and provides theoretical supporting to determination of the stereochemical relationships for complicated molecules.

**Figure 8:**
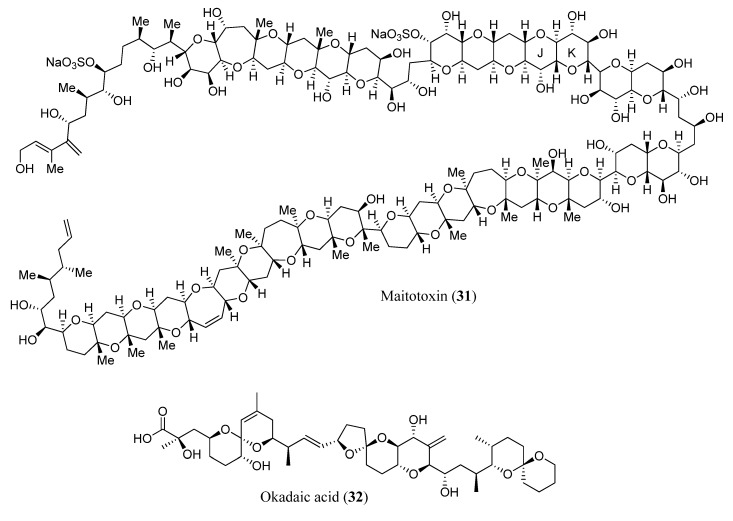
Original proposed structure of maitotoxin (**31**) and okadaic acid (**32**).

A renowned example of computational simulations of complicated compounds over the last few decades is the characterization of maitotoxin (**31**), which was isolated from *Gambierdiscus toxicus*, an armored, marine, benthic dinoflagellate species (Figure 8). The overall structure of maitotoxin, which contains 32 rings and 99 elements of stereochemistry (98 stereogenic centers and one trisubstituted double bond), and its absolute stereochemistry was proposed in a series of studies by the research groups of Yasumoto [62,63,64,65], Tachibana [66,67,68,69], and Kishi [70,71] between 1992 and 1996. Maitotoxin (**31**) was found to be the most potent and the largest known nonprotein substance. In 2006, Gallimore and Spencer questioned the stereochemistry at the J/K ring junction and implied that the C51 and C52 positions should have the opposite relative configuration [72]. The authors argue that this stereochemical regularity essentially arises from the assumed common biosynthetic origin of these structures, which involves the enzymatic epoxidation (from the same face) of polyunsaturated precursors followed by enzymatic openings of the epoxide with predictable stereochemical outcomes. Frederick *et al*., employed the program Spartan’06 with B3LYP/6-31G* DFT method to calculate the ^13^C-NMR chemical shifts of the truncated structures, which represent the three alternative maitotoxin structures under consideration [73]. As a result, the calculated values showed an excellent agreement with experimental ^13^C chemical shifts (average difference of 1.24 ppm), supporting that the originally proposed structure is most likely correct. The method was therefore considered a valuable tool to probe the structure discrepancies in this series of compounds, which includes maitotoxin.

In addition to maitotoxin, quantum mechanics computational simulations have been applied to structural characterization of the important group of polyether marine toxins okadaic acid (**32**) [56] (Figure 8), of which complex structures are usually elucidated on the basis of NMR spectral data [74,75,76]. In these reports, the effect of two different solvents, the use of two different levels of theory and the influence of molecular conformation on the ability of DFT calculations to predict the correct stereoisomer has been studied. The authors presented a systematic investigation of structure assignment using different statistical tools, such as correlation coefficient (*R*^2^), corrected mean absolute deviation (CMAD) and DP4 probability [56].

Stereochemistry is an important aspect of natural product research. The structures of marine natural products with stereochemical complexity have mostly been resolved by integrated chemical approaches that rely on detailed, painstakingly acquired data, sometimes over the course of several years, and often with limited sample sizes. Recent developments in spectroscopic techniques have enabled the identification of the structures of complex small molecules with microgram amount. However, there is no general solution to the absolute configuration of a compound even if it contains only one or few stereocenters.

**Figure 9:**
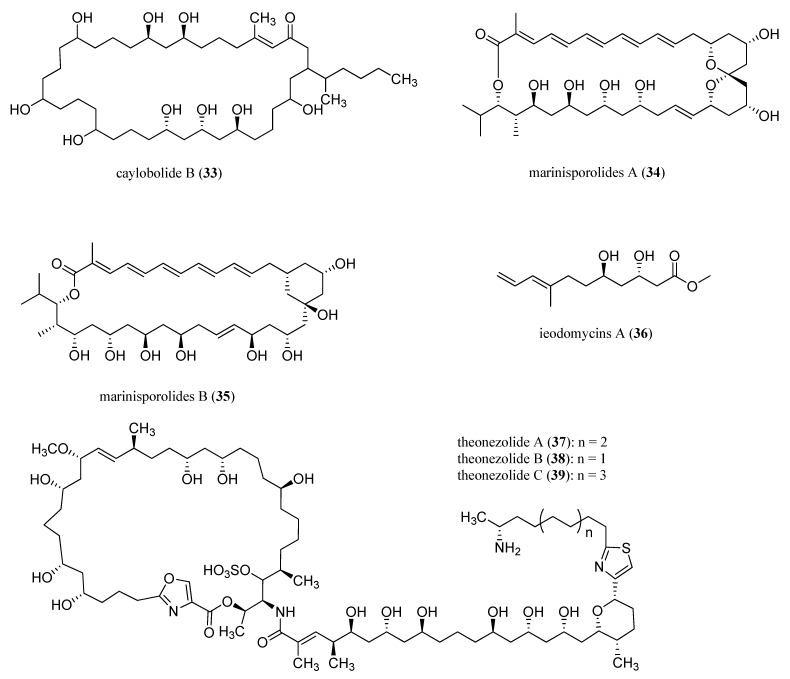
Caylobolide B (**33**), marinisporolides A and B (**34**–**35**), ieodomycin A (**36**), and theonezolides A–C (**37**–**39**).

Recently, integrated approaches to configuration assignment based on NMR database, chiro-optical, inferences from biosynthesis or bioinformatics, X-ray crystallography, and chemical synthesis has been widely applied to marine natural products [58]. Kishi and co-workers created a universal NMR database 1–5 for stereochemical assignment and applied to mycolactones [77,78,79,80,81,82,83]. Subsequently, this method was applied to the assignment of relative configuration for a new macrolactone derivative caylobolide B (**33**), isolated from a collection of *Phormidium* spp. [84], and two new polyene macrolides, marinisporolides A and B (**34**, **35**), isolated from a saline culture of the marine actinomycete (Figure 9) [85]. Kishi database 2 was applied to assign the relative configuration of the 3,5-diol of ieodomycins A (**36**) (Figure 9), an antimicrobial fatty acid isolated from a marine *Bacillus* sp. [86]. Moreover, Kishi database 1 and 2 were used as models to deduce the stereochemistry for the theonezolide A–C (**37**–**39**) (Figure 9), a variety of cytotoxic 37-membered macrolides with a long side chain isolated from an Okinawan marine sponge *Theonella* sp. [87].

It is particularly challenging to elucidate the relative and absolute configurations of multiple stereocenters with substantial overlaps in the methylene region. Their configuration assignment has greatly benefited from the development of Kishi’s Universal NMR Database as well as derivatization techniques, particularly Mosher’s analyses [88,89] and extension thereof [90,91]. However, applications of these methods are still under certain restrictions, particularly for those bearing 1,*n*-diol (*n* > 5) moieties. Assignment of the configuration of 1,*n*-diols has been demonstrated on model systems using exciton coupling circular dichroism after derivatization with arylcarboxylate chromophores within liposomes [92].

The determination of the absolute configurations of chiral molecules is an important aspect of molecular stereochemistry, and is often accomplished by comparison of electronic circular dichroism (ECD) and vibrational circular dichroism (VCD) with calculated values from density-functional theory (DFT). These techniques are useful in unambiguous assignment of absolute configurations in chiral molecules.

**Figure 10:**
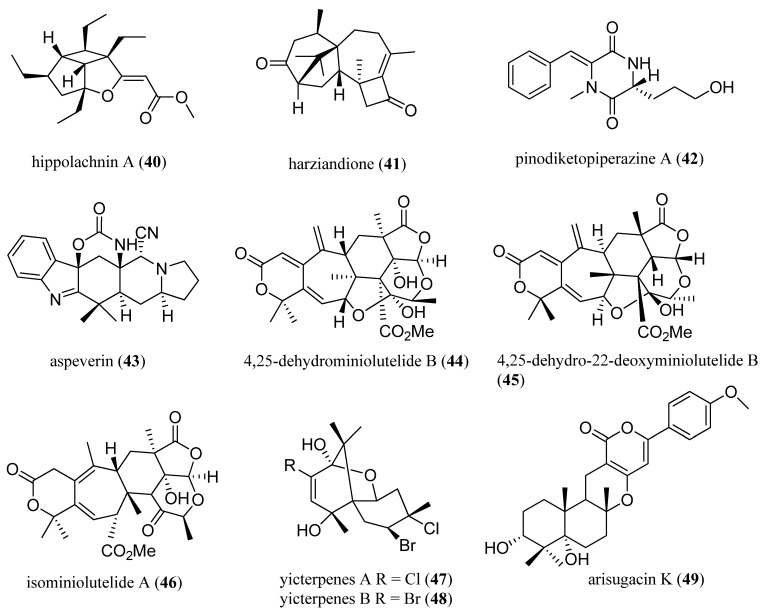
Hippolachnin A (**40**), harziandione (**41**), pinodiketopiperazine A (**42**), aspeverin (**43**), 4,25-dehydrominiolutelide B (**44**), 4,25-dehydro-22-deoxyminiolutelide B (**45**), and isominiolutelide A (**46**), yicterpenes A and B (**47**, **48**), and arisugacin K (**49**).

Recently, this method has been applied to assign absolute configurations of marine natural products. Hippolachnin A (**40**) (Figure 10) is a remarkable polyketide obtained from the South China sea sponge, *Hippospongia lachne*, by Lin and co-workers [93]. The planar structure, composed of three fused rings, in which an oxabicyclo-[3.3.0]-heptane further fused to a cyclobutane ring, was elucidated by a combination of NMR and MS analysis. The absolute configuration of hippolachnin A was assigned by calculating the ECD spectrum with TD-DFT and matching the measured and calculated spectra [93]. This method was applied by Ji and co-workers to assign absolute configurations for several marine natural products, including harziandione (**41**) [94], pinodiketopiperazine A (**42**) [95], aspeverin (**43**) [96], 4,25-dehydrominiolutelide B (**44**) [97], 4,25-dehydro-22-deoxyminiolutelide B (**45**) [97], isominiolutelide A (**46**) [97], yicterpenes A and B (**47**, **48**) [98], and arisugacin K (**49**) [99] (Figure 10).

**Figure 11:**
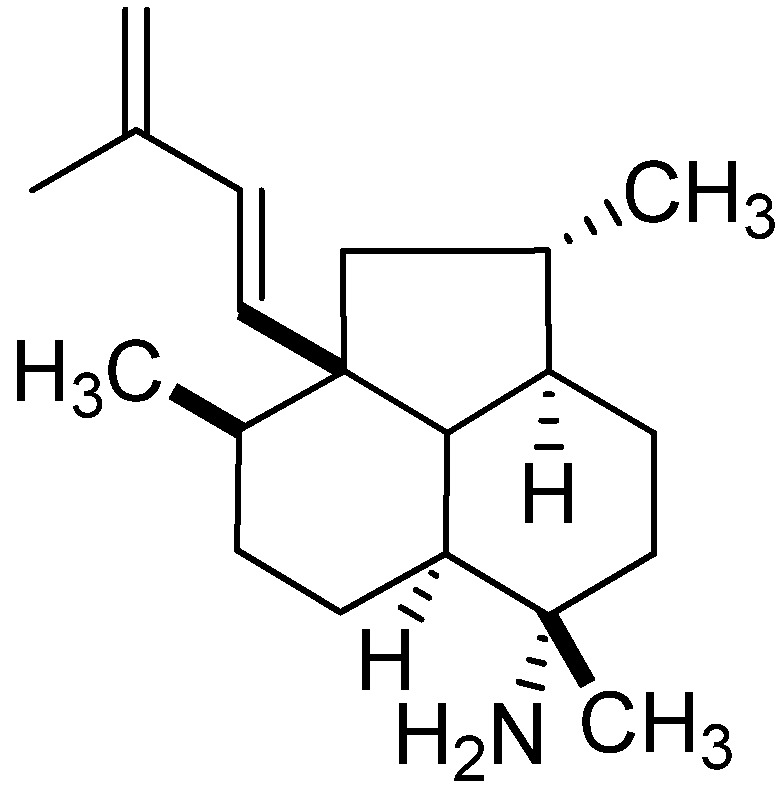
(1(14)-*E*,3S*,4R*,7S*,8S*,11R*,12S*,13R*)-7-formamidoisoneoamphilecta-1(14),15-diene (**50**).

Although less sensitive than ECD, VCD is gaining popularity for configuration assignment due to the ease of *ab initio* calculation of the theoretical vibronic spectra, especially in the “fingerprint region”, and no need for a chromophore. A notable example is the application of VCD for the assignment of absolute configuration of tricyclic diterpenes (1(14)-*E*,3S*, 4R*,7S*,8S*,11R*,12S*,13R*)-7-formamidoisoneoamphilecta-1(14),15-diene (**50**) (Figure 11), the primary amine derived from alkaline hydrolysis, after DFT calculation of the expected VCD spectra [100].

## 6. Concluding Remarks

The contribution of marine natural product research to the future pharmacopeia will likely become increasingly significant, with the development and further improvement of technologies for obtaining samples from extreme oceanic environments and charactering structures of trace amount molecules. New technologies and efficient collaborations between academic and industrial research will be essential to ensure the future success of marine natural products as new and novel therapeutic entities for the treatment of human disease.
